# Splenectomy modulates intrarenal B cell differentiation and impairs repair of post-ischemic kidney

**DOI:** 10.3389/fimmu.2025.1684731

**Published:** 2025-11-19

**Authors:** Kyungho Lee, Hojin Jeon, Kyung Sub Lee, Junseok Jeon, Jung Eun Lee, Ghee Young Kwon, Wooseong Huh, Hye Ryoun Jang

**Affiliations:** 1 Division of Nephrology, Department of Medicine, Samsung Medical Center, Cell and Gene Therapy Institute, Sungkyunkwan University School of Medicine, Seoul, Republic of Korea; 2 Samsung Advanced Institute for Health Sciences and Technology, Seoul, Republic of Korea; 3 Department of Pathology, Samsung Medical Center, Samsung Biomedical Research Institute, Sungkyunkwan University School of Medicine, Seoul, Republic of Korea

**Keywords:** acute kidney injury, B cell, immune cells, ischemia-reperfusion injury, repair, spleen, splenectomy

## Abstract

**Introduction:**

Lymphocytes are known to regulate kidney repair after ischemia-reperfusion injury (IRI). Splenectomy has been proposed as a preconditioning protocol for high-risk kidney transplantation and has been suggested to affect IRI outcomes. However, the role of splenectomy in IRI repair remains poorly understood. This study investigated the effects of splenectomy on the immunological microenvironment in a mouse model of kidney IRI.

**Methods:**

C57BL/6 mice underwent severe (45 min) unilateral (left) IRI and were divided into two groups: IRI surgery alone (IRI group) and IRI surgery with simultaneous splenectomy (IRI+SPX group). Post-ischemic and contralateral kidneys were collected on days 10 and 30 after IRI. Kidney function, histology, lymphocyte population (analyzed by flow cytometry), and cytokine/chemokine expression were evaluated.

**Results:**

The plasma creatinine levels were higher in the IRI+SPX group on day 10, while the cystatin C concentrations were not significantly different between the two groups. The percentage of tubular damage and fibrosis in post-ischemic kidneys during the repair phase was significantly higher in the IRI+SPX group than in the IRI group. While the T cell profiles were comparable between the groups, the proportions of activated B cells and MHCII+ B cells in the post-ischemic and contralateral kidneys were higher in the IRI+SPX group on day 30 after IRI. The expressions of IL-17, MCP-1, and TGF-β in post-ischemic kidneys were higher in the IRI+SPX group compared with the IRI group.

**Discussion:**

Splenectomy exacerbates tubular damage and fibrosis during the repair phase of severe IRI and significantly alters the immunological microenvironment of the kidneys, promoting B cell differentiation. Our study suggests that splenectomy may worsen outcomes in IRI, and further studies investigating potential reparative pathways through the kidney–spleen axis are warranted.

## 1 Introduction

Ischemia-reperfusion injury (IRI) is characterized by an initial restriction of blood supply, followed by restoration of perfusion, leading to oxidative stress, inflammation, and cellular damage (1). IRI is a major contributor to ischemic acute kidney injury (AKI) and an inevitable consequence of kidney transplantation that adversely affects transplant outcomes (2). Impaired recovery from IRI can result in kidney fibrosis and progression to chronic kidney disease (CKD). Among the various cellular and molecular pathways involved in the repair process after kidney IRI, lymphocyte-mediated immune responses are one of the key pathways (3). Sustained T cell responses in post-IRI kidneys play an important role in kidney repair or AKI to CKD transition (4). B cells have also been identified as the key mediators of kidney repair after IRI (5, 6). The trafficking of B cells into post-ischemic kidneys significantly affects both tubular atrophy and regeneration (5).

As lymphocyte-driven processes are increasingly recognized as therapeutic targets, the contribution of the spleen, an upstream regulator of systemic immunity, to IRI repair has become an area of particular interest (7, 8). Previous studies have shown that splenectomy can exert protective roles in acute phase of kidney IRI by ameliorating early inflammatory responses (9–13). Clinically, splenectomy was historically utilized as part of a preconditioning protocol to reduce B cells in ABO-incompatible kidney transplantations before the adoption of rituximab-based protocols (6, 7, 14). Splenectomy was also reported to reduce hyperacute rejection and improve graft survival (15). However, while the acute and early immunomodulatory effects of splenectomy have been studied, its impact on the recovery or repair after IRI remains unexplored.

Moreover, strategies aimed at suppressing inflammation during the injury processes may inadvertently disrupt the kidney repair process (3). Given that the spleen mediates cholinergic anti-inflammatory pathway, which plays a protective role in IRI through immune cells (16, 17), an intact spleen-kidney axis may be essential for adequate recovery after IRI. Since the spleen serves as a major reservoir of lymphocytes and splenectomy can alter systemic lymphocyte function, changes in kidney lymphocyte composition following splenectomy during repair phase after IRI warrant further investigation.

Therefore, investigating the impact of splenectomy on the repair process after kidney IRI is essential to understand how spleen-derived immune mechanisms affect kidney healing or fibrosis. In this study, we aimed to investigate the impact of splenectomy on the repair phase in a kidney IRI mouse model, focusing on kidney fibrosis, lymphocyte composition, and cytokine/chemokine expression in post-ischemic kidneys.

## 2 Materials and methods

### 2.1 Animals

C57BL/6 male mice at the age of 7 weeks were procured from Orient Bio Inc. (Seongnam, Kyoungki-do, Korea). The mice were housed under specific pathogen-free conditions at the Animal Facility of Samsung Medical Center. Mice were randomly allocated into two groups: IRI and IRI+splenectomy (IRI+SPX). This study was approved by the Institutional Review Board of Samsung Medical Center (IACUC no. 20180222002) and the Samsung Medical Center Animal Care and Use Committee.

### 2.2 Kidney IRI model

Established kidney IRI models were used as previously described (5, 18). Briefly, the mice were anesthetized with an intraperitoneal injection of ketamine (100mg/kg; Yuhan, Seoul, Korea) and xylazine (10mg/kg; Bayer, Leverkusen, Germany). Following a midline abdominal incision, the left renal pedicle was clamped for 45 minutes using microvascular clamps. During the procedure, mice were maintained at a constant body temperature (37°C) with a heating system and kept hydrated with warm sterile saline. Splenectomy was performed simultaneously while clamping the left renal pedicle. After the clamps were released, the wounds were sutured. The mice were provided with free access to food and water. For endpoint analyses, mice were euthanized on days 10 and 30 post-surgery under deep anesthesia with intraperitoneal ketamine (100mg/kg) and xylazine (10mg/kg) for organ collection. Mice were carefully exsanguinated prior to organ collection to remove circulating immune cells. Both post-ischemic and contralateral kidneys were collected for further analysis.

### 2.3 Assessment of kidney function

Blood samples were collected from the tail vein on days 0, 1, 3, 7, 10, 21, and 30 after surgery. The plasma creatinine (Cr) (Arbor Assays, Ann Arbor, MI, USA) and cystatin C (R&D Systems, Minneapolis, MN, USA) levels were measured.

### 2.4 Kidney histological analysis

Tissue sections were fixed with 10% formalin and subsequently stained with hematoxylin and eosin (H&E) or Masson’s trichrome. A renal pathologist, blinded to the experimental groups, performed quantitative histopathological analysis. Tubular damage was assessed on H&E-stained sections as the percentage of injured tubules relative to total tubules. Fibrosis was quantified on Masson’s trichrome-stained sections as the percentage of fibrotic area. A minimum of ten non-overlapping high-power fields per section were evaluated for both cortex and outer medulla separately. Data are presented as mean percentages across all analyzed fields.

### 2.5 CD45 immunohistochemistry of kidney tissues

To conduct immunohistochemistry (IHC) staining for CD45, tissue sections were first deparaffinized and rehydrated before being transferred to a citrate buffer solution (pH 6.0). The slides were placed in a pressure cooker and subjected to microwave heating for 10 min. They were then immersed in hydrogen peroxide solution (DAKO, Carpinteria, CA, USA) for 30 min and incubated overnight at 4 °C with a serum-free protein block (DAKO). The slides were then incubated for 1 h at room temperature with a 1:100 dilution of anti-mouse CD45 monoclonal antibody (BD Biosciences, San Jose, CA, USA). The staining process was completed by applying 3,3’-diaminobenzidine tetrahydrochloride and counterstaining with Mayer’s hematoxylin solution. Whole slide images were scanned and analyzed to quantify the percentage of CD45-positive cells relative to the total number of nucleated cells. Images were quantified using the positive cell detection tool in QuPath software (version 0.5.0) (**Supplementary Figure S1**) (19).

### 2.6 Flow cytometry analysis of kidney mononuclear cells

Kidney mononuclear cells (KMNCs) were isolated according to a previously established Percoll density gradient technique (20). Briefly, decapsulated kidneys were suspended in RPMI buffer (Mediatech, Manassas, VA) containing 5% fetal bovine serum and mechanically disrupted using a Stomacher 80 Biomaster (Seward, Worthing, West Sussex, UK). The resulting cell suspension was passed through 70-µm cell strainers (BD Biosciences), washed, and resuspended in 36% Percoll (Amersham Pharmacia Biotech, Piscataway, NJ). The cell suspension was then carefully layered over 72% Percoll. The samples were centrifuged at 1,000 × *g* for 30 min at room temperature, and KMNCs were collected from the interface between the 36% and 72% Percoll layers. The number of viable KMNCs was determined using an automated cell counter (Life Technologies, Carlsbad, CA, USA).

The isolated KMNCs were resuspended in fluorescence-activated cell sorting (FACS) buffer and pre-incubated with anti-CD16/32 antibodies (BD Biosciences) for 10 min to minimize nonspecific antibody binding. KMNCs were then incubated with anti-mouse APC anti-CD3 (17A2), APC TCRβ (H57-597), PerCp anti-CD4 (GK 1.5), FITC ani-CD8 (16-10A1), PerCp anti-CD19 (1D3), BV510 anti-CD21 (7G6), APC-Cy7 anti-CD69 (H1.2F3), PE anti-CD25 (3C7), PE-Cy7 anti-CD27(LG.3A10), PE anti-CD126 (D7715A7), APC anti-CD138 (281-2), BV421 anti-FoxP3 (MF23), and FITC MHC II (25-9-17) (all from BD Biosciences) for 25 minutes at 4°C, followed by washing with FACS buffer. Stained KMNCs were analyzed using a FACSVerse flow cytometer (BD Biosciences) and FlowJo software (version 10.10.0; BD Biosciences).

### 2.7 Multiplex cytokine/chemokine assay

Cytokines and chemokines were measured in kidney protein extracts using a multiplexed, particle-based, flow cytometric assay with a Milliplex MAP Mouse Cytokine/Chemokine Kit (Luminex, Austin, TX, USA) and Mouse Magnetic Luminex Assay Kit (R&D Systems, Minneapolis, MN, USA) according to the manufacturer’s instructions. Interleukin (IL)-6, IL-10, IL-17, interferon-gamma (INF)-γ, monocyte chemoattractant protein (MCP)-1, TNF-α, regulated on activation, normal T cell expressed and secreted (RANTES, as known as chemokine C-C motif ligand 5, CCL5), and vascular endothelial growth factor (VEGF) were analyzed on day 10 post-IRI. Intrarenal expression of tumor growth factor (TGF)-β was measured on day 30 post-IRI using a quantikine kit (R&D Systems). To normalize the cytokine and chemokine concentrations, raw protein concentrations were measured using the Pierce BCA Protein Assay Kit (Thermo Fisher Scientific, Waltham, MA, USA).

### 2.8 Statistical analysis

The results are expressed as the mean ± standard deviation (SD). Differences between groups were analyzed at each time point using the Mann-Whitney U test. All statistical analyses were performed using GraphPad Prism 10 software (San Diego, CA, USA). Statistical significance was determined as p-value < 0.05 in a two-tailed test.

## 3 Results

### 3.1 Changes in kidney function following unilateral IRI

**Figure 1:**
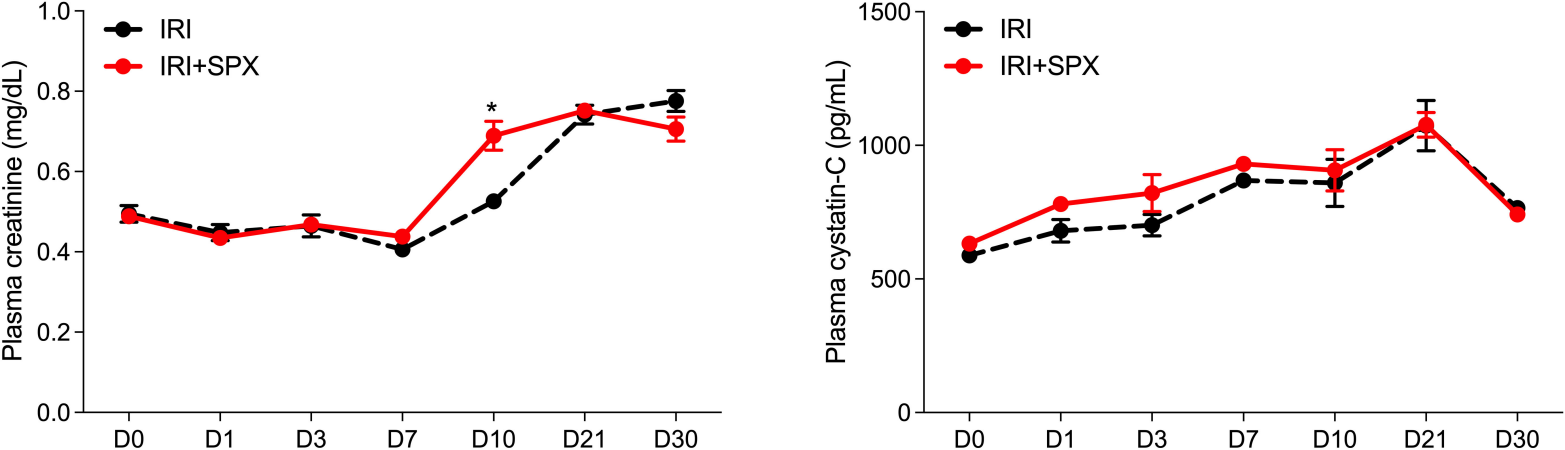
Serial follow-ups of kidney function after IRI. Plasma creatinine levels were measured using an enzymatic assay, and cystatin C levels were measured by ELISA. Samples were collected on days 3, 10, and 30 after surgery. Data are presented as mean ± SD (n=5 per group per time point). Statistical analysis was performed using the Mann-Whitney U test. P < 0.05 was considered statistically significant. IRI, ischemia-reperfusion injury; SPX, splenectomy.

The plasma Cr levels were higher at day 10 in the IRI+SPX group than in the IRI group (IRI: 0.53 ± 0.02 vs. IRI+SPX: 0.69 ± 0.04 mg/dL, *P* < 0.001), returning to comparable levels from 3 weeks after IRI. The cystatin C concentrations were not significantly different between the IRI and IRI+SPX groups during the first week following IRI (**Figure 1**).

### 3.2 Splenectomy exacerbated tubular damage and fibrosis during post-IRI repair

**Figure 2:**
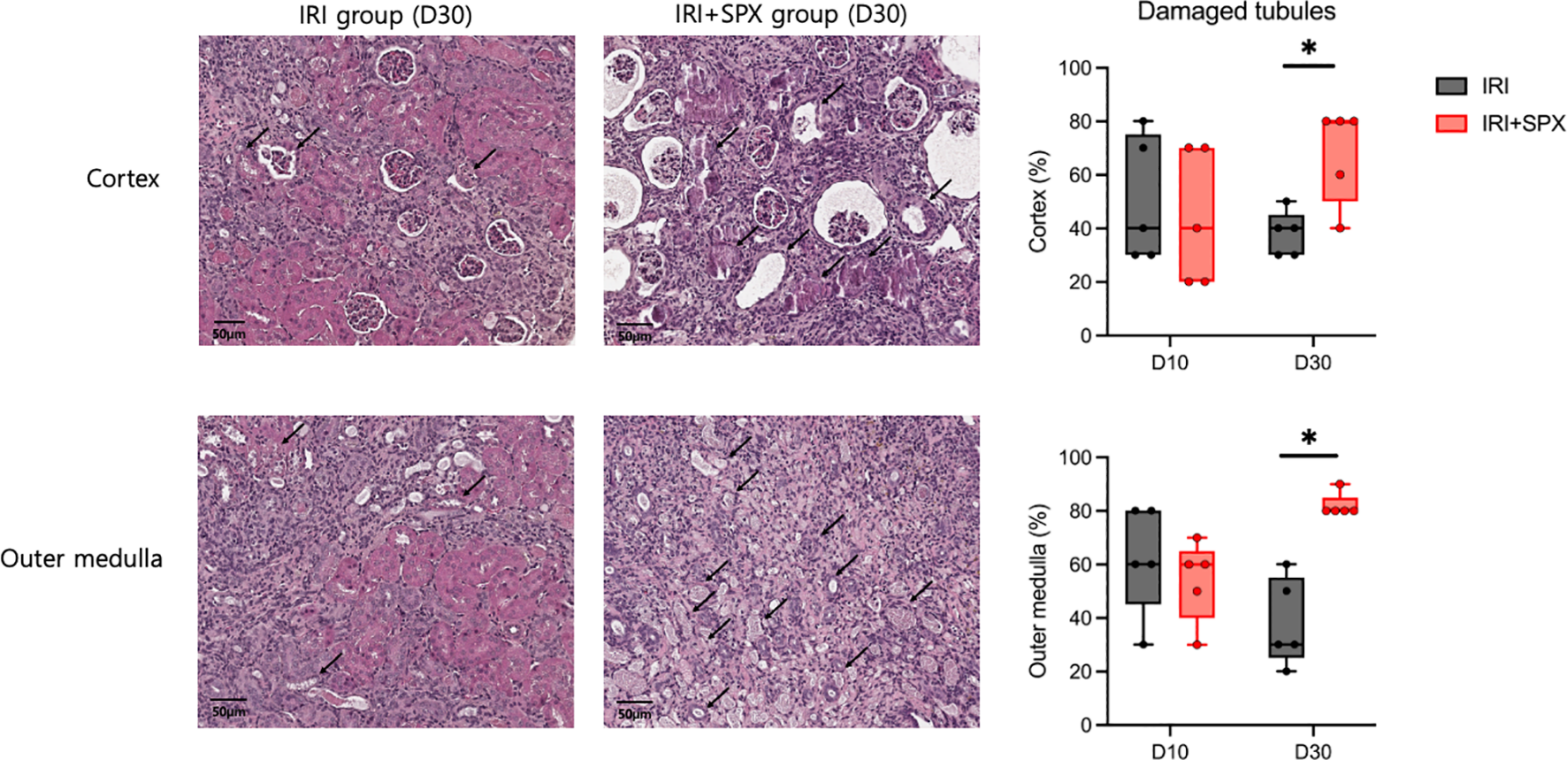
H&E staining of post-ischemic kidneys in the IRI and IRI+SPX groups. Histological analysis of tubular injury in post-ischemic kidneys using H&E staining. Representative images and quantitative analysis of tubular damage and atrophy in the cortex and outer medulla are shown. Quantification was based on blinded scoring of 10 randomly selected high-power fields per kidney. Post-ischemic kidneys were collected on days 10 and 30 post-IRI. Data are presented as mean ± SD (n=5 per group). Statistical comparisons were made using the Mann-Whitney U test. P < 0.05 was considered statistically significant. IRI, ischemia-reperfusion injury; SPX, splenectomy. * P < 0.05.

**Figure 3:**
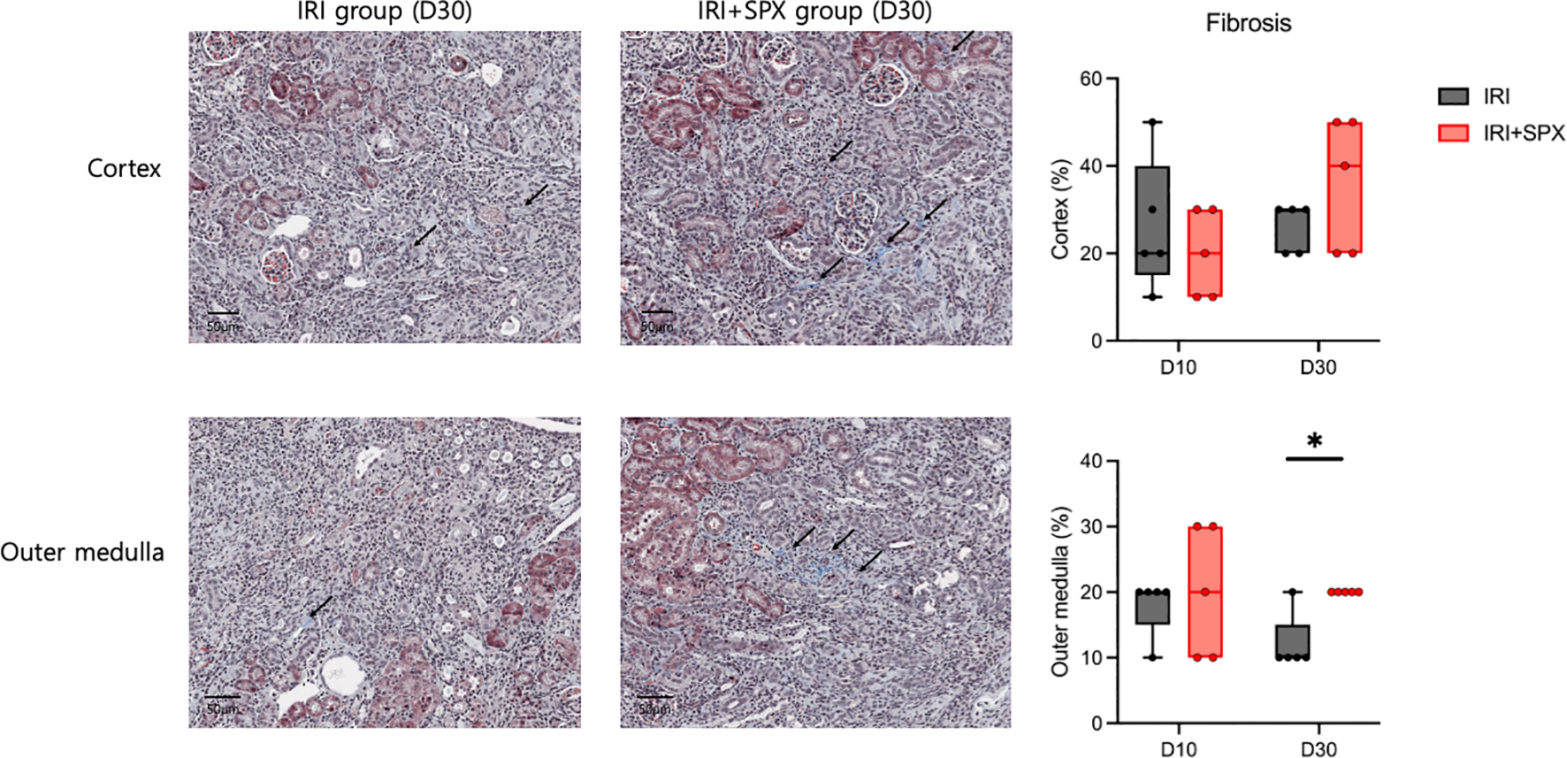
Fibrosis in the outer medulla of post-ischemic kidneys in the IRI and IRI+SPX groups. Assessment of fibrosis in post-ischemic kidneys using Masson’s trichrome staining. Fibrotic area (%) was quantified by image analysis of 10 randomly selected fields per kidney in the outer medulla. Kidneys were collected on days 10 and 30 after IRI. Data are shown as mean ± SD (n=5 per group). Statistical significance was determined by Mann-Whitney U test. P < 0.05 was considered statistically significant. * P < 0.05.

To study how splenectomy affects tubular atrophy and fibrosis, kidney sections stained with H&E and Masson’s trichrome were studied at 10 and 30 days after IRI. H&E staining of the post-ischemic kidneys in both groups revealed significant tubular damage, inflammatory cell infiltration, and tubular atrophy after IRI (**Figure 2**). Ten days after IRI, the IRI+SPX group exhibited comparable degrees of tubular damage and fibrosis in the cortex and outer medulla to the IRI group. However, 30 days after IRI, the percentage of damaged tubules in the cortex and outer medulla was significantly higher in the IRI+SPX group than in the IRI group. Additionally, the degree of fibrosis in the outer medulla was higher in the IRI+SPX group than that in the IRI group (**Figure 3**).

### 3.3 Splenectomy increased chronic leukocyte infiltration in post-IRI kidneys during repair

**Figure 4:**
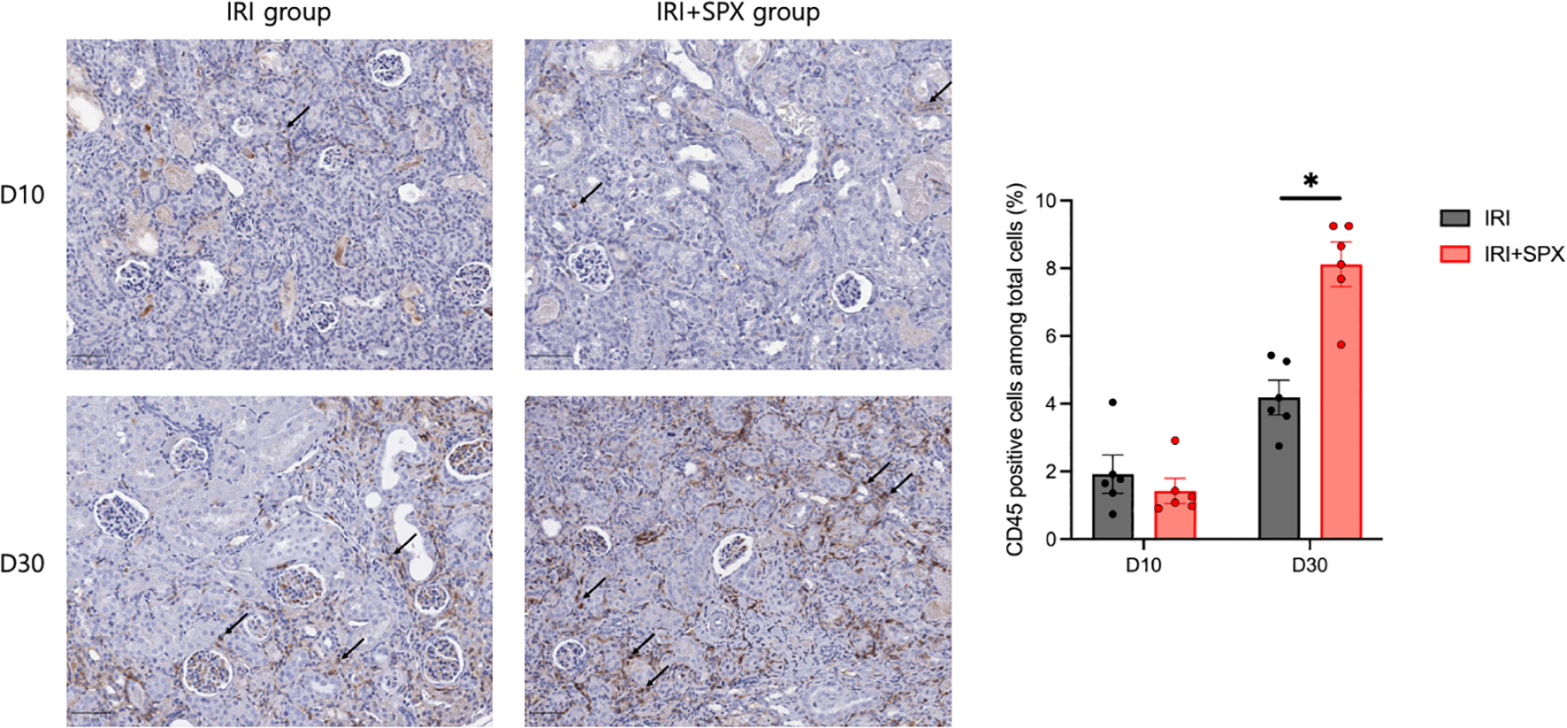
Intrarenal trafficking of leukocytes in post-ischemic kidneys in the IRI and IRI+SPX groups. Intrarenal trafficking of total leukocytes (CD45+ cells) in post-ischemic kidneys. Immunohistochemistry for CD45 was performed, and positive cells were quantified as a percentage of total nucleated cells using automated image analysis. Kidneys were analyzed on days 10 and 30 post-IRI. Data represent mean ± SD (n=5 per group). Statistical analysis was conducted using the Mann-Whitney U test. P < 0.05 was considered statistically significant. * P < 0.05.

To investigate the effect of splenectomy on chronic infiltration of leukocytes, the amount of total leukocytes expressing CD45 from the kidney sections was quantified using an automated imaging analysis system in post-ischemic kidneys (**Supplementary Figure S1**). The percentage of leukocytes relative to the total number of nucleated cells increased after IRI in both groups. After 30 days post-IRI, the percentage of intrarenal leukocytes was higher in the IRI+SPX group compared with the IRI group (IRI: 4.18 ± 0.51 vs. IRI+SPX: 8.12 ± 0.66%, *P* < 0.001) (**Figure 4**).

### 3.4 Splenectomy shifted kidney B cells toward activated and memory phenotypes during post-IRI repair

**Figure 5:**
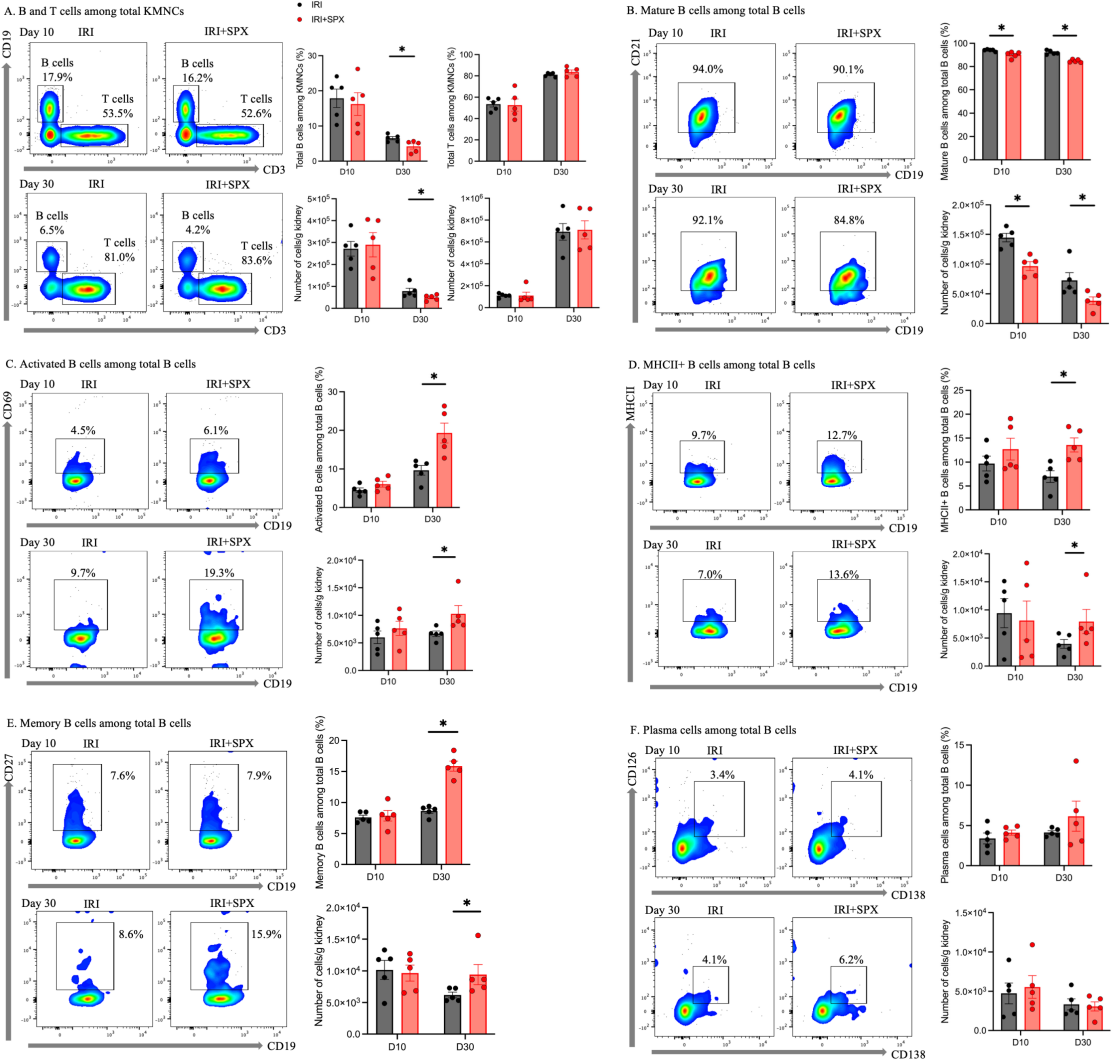
Analysis of B cell subtypes in post-ischemic kidneys on days 10 and 30 post-IRI in the IRI and IRI+SPX groups. Flow cytometric analysis of B cell subsets in post-ischemic kidneys. Single-cell suspensions were prepared from kidneys, and immune cell populations were analyzed by flow cytometry. B cell subsets included **(A)** total B cells (CD19+), **(B)** mature B cells (CD21+), **(C)** activated B cells (CD69+), **(D)** MHCII+ B cells, **(E)** memory B cells (CD27+), and **(F)** plasma cells (CD138+, CD126+). Both percentages and absolute counts of B subsets were analyzed. While compositional differences were reflected in percentages, absolute cell counts provided complementary quantitative data for each subset. Measurements were taken on days 10 and 30 after IRI. Data are expressed as mean ± SD (n=5 per group). Statistical significance was assessed by Mann-Whitney U test. P < 0.05 was considered statistically significant. * P < 0.05.

We analyzed kidney mononuclear cells in post-IRI kidneys to study the effect of splenectomy on kidney lymphocytes. The gating strategies for kidney lymphocytes are provided in **Supplementary Figure S2**. The proportions of B cell subtypes in the post-ischemic and contralateral kidneys were analyzed 10 and 30 days after IRI (**Figure 5**, **Table 1**, **Supplementary Table S1**). In the post-ischemic kidneys, the proportion of total B cells expressing CD19 among KMNCs gradually decreased over time in both groups (day 10 and 30; IRI, 17.88 ± 5.84 and 6.53 ± 1.03%, *P* < 0.001; IRI+SPX, 16.24 ± 7.20, and 4.21 ± 1.68%, *P* < 0.001). On day 30 after IRI, the proportion of total B cells was lower in the IRI+SPX group than in the IRI group (*P* = 0.032) (**Figure 5A**). The proportion of mature B cells expressing CD21 among the total B cells was lower in the IRI+SPX group on both days 10 and 30 than in the IRI group (day 10, IRI: 94.04 ± 0.41 vs. IRI+SPX: 90.12 ± 1.20, *P* = 0.008/day 30, IRI: 92.10 ± 0.78 vs. IRI+SPX: 84.78 ± 0.56, *P* = 0.008) (**Figure 5B**). The proportions of activated B cells expressing CD69, MHCII+ B cells, and memory B cells expressing CD27 among total B cells were higher in the IRI+SPX group compared with the IRI group on day 30 after IRI (activated B cells, IRI: 9.66 ± 2.78 vs. IRI+SPX: 19.30 ± 5.76%, *P* = 0.008/MHCII+ B cells, IRI: 6.97 ± 2.76 vs. IRI+SPX: 13.58 ± 3.26%, *P* = 0.008/memory B cells, IRI: 8.64 ± 0.86 vs. IRI+SPX: 15.86 ± 1.81%, *P* = 0.008) (**Figures 5C–E**). The proportion of plasma cells expressing CD138 and CD126 among total B cells was comparable between the IRI and IRI+SPX groups (**Figure 5F**).

**Table 1:** B cell populations in post-ischemic and contralateral kidneys.

% of cells in post-ischemic kidneys	Day 10 after IRI			Day 30 after IRI		
	IRI	IRI+SPX	p-value	IRI	IRI+SPX	p-value
Total B cells	17.88 ± 5.84	16.24 ± 7.20	0.643	6.53 ± 1.03	4.21 ± 1.68	0.032
Activated B cells	4.51 ± 1.29	6.07 ± 1.67	0.151	9.66 ± 2.78	19.30 ± 5.76	0.008
MHCII+ B cells	9.70 ± 3.43	12.69 ± 5.08	0.548	6.97 ± 2.76	13.58 ± 3.26	0.008
Mature B cells	94.04 ± 0.91	90.12 ± 2.69	0.008	92.10 ± 1.74	84.78 ± 1.25	0.008
Memory B cells	7.61 ± 0.83	7.88 ± 1.89	0.841	8.64 ± 0.86	15.86 ± 1.81	0.008
Plasma cells	3.40 ± 1.53	4.12 ± 0.73	0.548	4.14 ± 0.50	6.16 ± 4.19	0.690
% of cells in contralateral kidneys	Day 10 after IRI			Day 30 after IRI		
	IRI	IRI+SPX	p-value	IRI	IRI+SPX	p-value
Total B cells	32.92 ± 3.09	22.56 ± 4.42	0.008	29.96 ± 6.66	15.38 ± 2.18	0.008
Activated B cells	2.24 ± 1.12	2.55 ± 0.85	0.841	1.65 ± 0.52	3.46 ± 0.49	0.008
MHCII+ B cells	7.43 ± 1.28	11.72 ± 3.76	0.095	9.47 ± 2.37	18.16 ± 1.61	0.008
Mature B cells	91.74 ± 1.88	88.38 ± 4.54	0.278	94.18 ± 1.01	88.94 ± 2.74	0.008
Memory B cells	7.05 ± 1.49	7.35 ± 0.86	0.841	9.06 ± 1.95	9.83 ± 1.77	0.524
Plasma cells	0.62 ± 0.24	0.27 ± 0.14	0.032	0.45 ± 0.15	0.21 ± 0.10	0.032

Values represent the mean ± SEM of percentages of gated cells; n = 5/group. KMNCs, kidney mononuclear cells (KMNCs) expressing CD45 of lymphocyte gating on the FSC vs. SSC plot; total B cells expressing CD19 among total KMNCs; activated B cells expressing both CD69 and CD21 among total B cells; MHC II+ B cells expressing MHC II among total B cells; mature B cells expressing CD21 among total B cells; memory B cells expressing CD27 among total B cells; and plasma cells expressing both CD138 and CD126 among total B cells.

IRI, ischemia-reperfusion injury; SPX, splenectomy.

**Figure 6:**
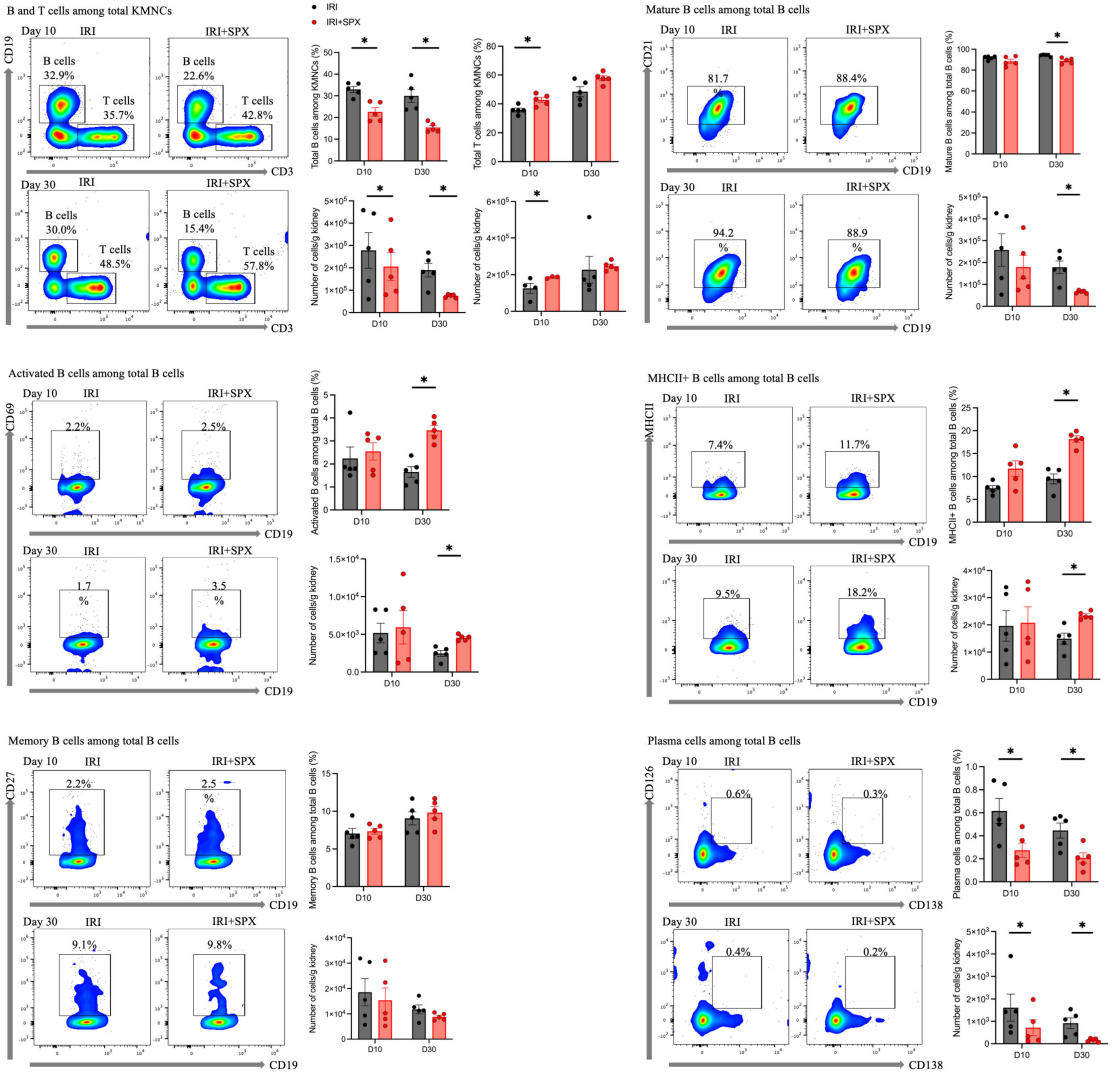
Analysis of B cell subtypes in contralateral kidneys on days 10 and 30 post-IRI in the IRI and IRI+SPX groups. Flow cytometric analysis of B cell subsets in contralateral kidneys. Immune cell profiling was performed as described for post-ischemic kidneys. Both percentages and absolute counts of B subsets were analyzed. Samples were collected on days 10 and 30 post-IRI. Data are presented as mean ± SD (n=5 per group). Differences between groups were analyzed using the Mann-Whitney U test. P < 0.05 was considered statistically significant. * P < 0.05.

In the contralateral kidneys (**Figure 6**, **Table 1**), the proportion of total B cells among KMNCs in the IRI+SPX group was lower than that in the IRI group at all time points. The proportion of mature B cells among total B cells was lower in the IRI+SPX compared with the IRI group on day 30 after IRI (IRI: 94.18 ± 1.01 vs. IRI+SPX: 88.94 ± 2.74%, *P* = 0.008) (**Figure 6B**). The proportions of activated B cells and MHCII+ B cells among total B cells were higher in the IRI+SPX group compared with the IRI group on day 30 after IRI (activated B cells, IRI: 1.65 ± 0.52 vs. IRI+SPX: 3.46 ± 0.49%, *P* = 0.008/MHCII+ B cells, IRI: 9.47 ± 2.37 vs. IRI+SPX: 18.16 ± 1.61%, *P* = 0.008) (**Figures 6C, D**). The proportion of memory B cells was comparable between the two groups (**Figure 6E**). The proportion of plasma cells among total B cells was lower in the IRI+SPX group (day 10, IRI: 0.62 ± 0.24 vs. IRI+SPX: 0.27 ± 0.14%, *P* = 0.032/day 30, IRI: 0.45 ± 0.15 vs. IRI+SPX: 0.21 ± 0.10%, *P* = 0.032) (**Figure 6F**).

### 3.5 Splenectomy did not significantly alter T cell phenotypes in post-IRI kidneys during repair

The proportions of T cell subtypes in the post-ischemic and contralateral kidneys were also analyzed 10 and 30 days after IRI (**Table 2**, **Supplementary Figure S4**, **Supplementary Table S2**). In post-ischemic kidneys, the proportion of total T cells among KMNCs increased over time (day 10 and 30; IRI, 53.50 ± 5.90 and 81.02 ± 1.76%, *P* < 0.001; IRI+SPX, 52.62 ± 12.7 and 83.62 ± 4.36%, *P* < 0.001) (**Figure 5A**). The proportions of CD4+ and CD8+ T cells among the total T cells were comparable between the two groups (**Supplementary Figure S4**). The proportion of regulatory T cells (Tregs) increased over time and was comparable between the two groups (day 10, IRI: 0.29 ± 0.20 vs. IRI+SPX: 0.35 ± 0.22, *P* = 0.523/day 30, IRI: 0.42 ± 0.14 vs. IRI+SPX: 0.60 ± 0.30, *P* = 0.222) (**Supplementary Figure S4**).

**Table 2:** T cell populations in post-ischemic and contralateral kidneys.

% of cells in post-ischemic kidneys	Day 10 after IRI			Day 30 after IRI		
	IRI	IRI+SPX	p-value	IRI	IRI+SPX	p-value
Total T cells	53.50 ± 5.90	52.62 ± 12.7	0.999	81.02 ± 1.76	83.62 ± 4.36	0.421
CD8 T cells	27.28 ± 1.76	21.46 ± 4.56	0.063	17.82 ± 4.28	22.92 ± 3.89	0.056
CD4 T cells	65.14 ± 3.64	67.86 ± 9.54	0.548	76.46 ± 4.08	71.02 ± 3.76	0.056
Treg cells	0.29 ± 0.20	0.35 ± 0.22	0.523	0.42 ± 0.14	0.60 ± 0.30	0.222
% of cells in contralateral kidneys	Day 10 after IRI			Day 30 after IRI		
	IRI	IRI+SPX	p-value	IRI	IRI+SPX	p-value
Total T cells	35.70 ± 2.95	42.76 ± 3.92	0.023	48.46 ± 7.67	57.76 ± 3.92	0.063
CD8 T cells	32.43 ± 0.98	30.33 ± 3.21	0.629	34.06 ± 1.86	33.22 ± 1.63	0.548
CD4 T cells	61.75 ± 0.70	66.07 ± 3.66	0.143	61.28 ± 1.22	61.50 ± 1.32	0.841
Treg cells	0.21 ± 0.07	0.23 ± 0.07	0.714	0.32 ± 0.09	0.37 ± 0.23	0.897

Values represent the mean ± SEM of percentages of gated cells; n = 5/group. KMNCs: kidney mononuclear cells (KMNCs) expressing CD45 of lymphocyte gating on the FSC vs. SSC plot; total T cells expressing CD3 among total KMNCs; CD8 T cells expressing CD8 among total T cells; CD4 T cells expressing CD4 among total T cells; regulatory T (Treg) cells expressing both FoxP3 and CD25 among total T cells.

IRI, ischemia-reperfusion injury; SPX, splenectomy.

In the contralateral kidneys (**Table 2**), the proportion of total T cells among KMNCs was higher in the IRI+SPX group than in the IRI group on day 10 after surgery (IRI: 35.70. ± 2.95 vs. IRI+SPX: 42.76 ± 3.92%, *P* = 0.023) (**Figure 6A**). The proportions of CD4 and CD8 T cells, as well as Tregs, were comparable between the groups (**Supplementary Figure S4**).

Further analyses of CD4+ T cell subsets revealed no significant differences in activated or effector memory phenotypes between the IRI and IRI+SPX groups, while effector memory CD8+ T cells increased in the post-ischemic kidneys of the IRI+SPX group on day 30 (**Supplementary Table S3**).

### 3.6 Splenectomy altered kidney cytokine production during post-IRI repair

**Figure 7:**
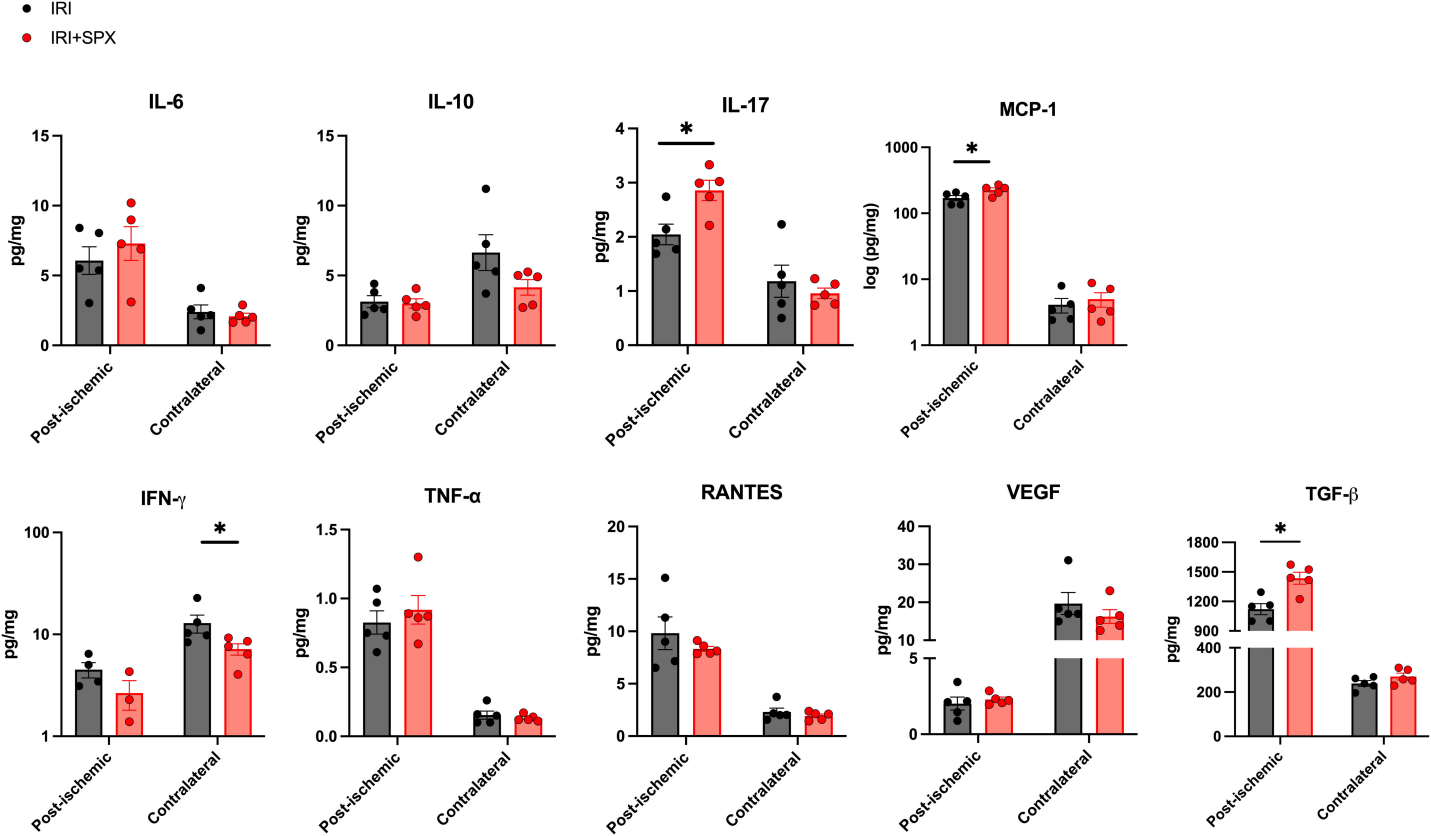
Expression levels of cytokines and chemokines in post-ischemic and contralateral kidneys in the IRI and IRI+SPX groups. Expression levels of cytokines and chemokines in post-ischemic and contralateral kidneys. IL-17, MCP-1, and TGF-β were significantly higher in the post-ischemic kidneys from the IRI+SPX group. Data are shown as mean ± SD (n=5 per group). Statistical analysis was performed using the Mann-Whitney U test. P < 0.05 was considered statistically significant. IL, interleukin; INF, interferon; MCP, monocyte chemoattractant protein; RANTES, regulated on activation, normal T cell expressed and secreted; TGF, transforming growth factor; TNF, tumor necrosis factor; VEGF, vascular endothelial growth factor. * P < 0.05.

We measured cytokine and chemokine expression during repair after IRI to study how splenectomy affects inflammatory and fibrotic milieu. The expression levels of cytokines and chemokines in the post-ischemic and contralateral kidneys are shown in **Figure 7**. The expression of IL-17, MCP-1, and TGF-β was higher in the IRI+SPX group compared with the IRI group (IL-17, IRI: 2.05 ± 0.42 vs. IRI+SPX: 2.86 ± 0.42 pg/mg, *P* = 0.024/MCP-1, IRI: 170.77 ± 33.65 vs. IRI+SPX: 226.71 ± 38.43 pg/mg, *P* = 0.040/TGF-β, IRI: 1122 ± 124.1 vs. IRI+SPX: 1436 ± 134.7 pg/mg, *P* = 0.016). The levels of other cytokines were comparable between groups.

The expression of IFN-γ was lower in the IRI+SPX group compared with the IRI group in contralateral kidneys (IRI: 12.89 ± 2.60 vs. IRI+SPX: 7.18 ± 0.92 pg/mg, *P* = 0.032).

## 4 Discussion

This study demonstrates that splenectomy impairs tubular regeneration and exacerbates interstitial fibrosis in post-ischemic kidneys during the IRI repair phase. Although the proportion of T-cell subtypes was comparable between the two groups, splenectomy promoted intrarenal B-cell differentiation. Furthermore, the expression of inflammatory and pro-fibrotic cytokines, including IL-17, MCP-1, and TGF-β, were upregulated by splenectomy in post-ischemic kidneys. These findings suggest that the spleen may contribute to kidney repair not only by regulating systemic immune responses but also by modulating kidney B cell responses in the post-IRI kidneys. The enhanced activation and differentiation of B cells following splenectomy may promote a pro-fibrotic microenvironment through the secretion of proinflammatory and profibrotic cytokines/chemokines. Thus, the disruption of the spleen-kidney axis appears to shift the immunologic milieu toward a maladaptive and fibrotic response in IRI.

Given the limited understanding of the role of splenectomy in repair after IRI, we focused on renal histological and immunological changes beyond 10 days post-IRI with splenectomy. Pronounced leukocyte trafficking, severe tubular atrophy, and interstitial fibrosis were observed in the splenectomy group, suggesting that splenectomy is likely to increase kidney leukocyte expansion and promote inflammation during the recovery phase of IRI. Several studies have investigated the role of splenectomy in the early phase of kidney IRI. Splenectomy appears to have a contrasting protective role during the early phase of AKI. In a rat IRI model, splenectomy reduced BUN and serum Cr levels as well as histological damage 24 h after reperfusion (9). Additionally, splenectomy reduced macrophage/monocyte infiltration and the levels of TNF-α and IL-6 at 24 hours after reperfusion (11). Taken together, splenectomy is likely to have a dual nature in renal IRI, providing an anti-inflammatory effect in the early phase but contributing to a pro-fibrotic effect in the later repair phase. This underscores the risk that strategies targeting early inflammatory processes may inadvertently impair renal tubular regeneration (2). Although splenectomy was performed simultaneously with IRI in the present study, varying the timing of the splenectomy (before or after IRI) might have led to different outcomes. Further studies investigating splenectomy at different time points are warranted.

An earlier study demonstrated that mature B cells infiltrate post-IRI kidneys until four weeks after IRI (5). This late B cell infiltration into post-IRI kidneys was thought to be involved in kidney fibrosis and the AKI-to-CKD transition (5, 21). We found that the differentiation of B cells into MHC II+ and memory B cells was higher in the IRI+SPX group than in the IRI group, which could explain the pronounced fibrosis following IRI with splenectomy. Future mechanistic studies using B cell-deficient mice or B cell depleting techniques in splenectomy models are warranted to further elucidate the role of B cells in modulating the spleen-kidney axis following IRI. Moreover, germinal center B cells expressing MHC II and memory B cells expressing CD27 are typically found in the spleen (22). It could be speculated that IRI in the setting of splenectomy might have induced the formation of lymphoid tissues in the injured kidneys, promoting the differentiation of B cells. Tertiary lymphoid tissue (TLTs) is an ectopic lymphoid structure that develops under chronic inflammatory conditions (23–25). TLTs, ectopic lymphoid tissues composed of mostly T and B cells and stromal cells, have been found in both human and mouse kidney allograft tissues and are associated with a higher risk of graft dysfunction (26, 27). Increased proportions of MHC II+ B cells and memory B cells in the IRI+SPX group may have contributed to the formation of lymphoid tissues and sustained inflammation during the repair phase of IRI. However, this needs to be confirmed in future studies that specifically examine TLTs in post-ischemic kidneys. Interestingly, activated B cell phenotypes increased not only in post-ischemic kidneys but also in contralateral kidneys. This finding suggests that IRI triggers systemic immune activation, likely through circulating damage-associated molecular patterns and cytokines, and that splenectomy further perturbs B cell homeostasis, leading to their redistribution and activation in uninvolved kidneys. Furthermore, in addition to canonical B cells, future study including in-depth B cell markers, such as extrafollicular B cells, is warranted to further understand the role of B cells in IRI.

Splenectomy led to the upregulation of IL-17, MCP-1, and TGF-β in post-ischemic kidneys, suggesting a potential link between promoted B cell differentiation and increased fibrosis. Kidney lymphocyte-derived IL-17 contributes to kidney fibrosis and AKI-to-CKD transition after IRI (28–30). This cytokine also promotes the expression of CXCL12, which facilitates the recruitment of B cells and development of B cell follicles (31–33). MCP-1, a proinflammatory chemokine, is secreted by various immune and non-immune cells. Among these, B cells appear to be the major source of renal MCP-1 (34). MCP-1 plays a role in B cell-mediated monocyte and macrophage infiltration, and blocking MCP-1 can reduce macrophage infiltration and renal injury (35). TGF-β promotes fibrosis and tubulointerstitial damage by upregulating pro-fibrotic genes and activating pathways that reduce cell proliferation and enhance apoptosis in AKI (36, 37). Notably, IFN-γ levels were lower in the contralateral kidneys of the IRI+SPX group compared with the IRI group. This finding may reflect the loss of the spleen as a major reservoir of IFN-γ-producing NK and T cells, resulting in diminished systemic IFN-γ availability for remote organs. This finding highlights that splenectomy alters systemic immune mediator distribution beyond the primary injury site. However, the precise mechanism was not addressed in this study and warrants further investigation.

There are several other plausible mechanisms that may explain the deleterious effects of splenectomy on IRI repair. Considering that the spleen mediates kidney protection through the cholinergic anti-inflammatory pathway (16, 17), the loss of the intact cholinergic anti-inflammatory pathway in splenectomized mice may have contributed to increased kidney fibrosis and inflammation. Splenic IL-10 production downregulates proinflammatory responses to IRI (38). Unlike conventional B cells that play pro-fibrotic roles, IL-10-producing B cells, typically classified as regulatory B cells, suppress chronic inflammation (5). Since no differences in the regulatory T cell subset based on the splenectomy status were observed, the absence of splenic IL-10 and regulatory B cells may have contributed to the persistent inflammation and subsequent kidney fibrosis observed in the splenectomy group. However, these findings will need to be verified in future studies using regulatory B cell-specific markers. The potential regulatory and repair roles of the spleen in IRI warrant further investigation.

Our study has some limitations. First, although we focused on B and T cell subtypes, other types of intrarenal immune cells, such as macrophages, dendritic cells, neutrophils, and innate lymphoid cells, could also play important roles in IRI repair (3). The pathogenic role of B cells appears to involve orchestrating the recruitment of innate immune cells, as demonstrated by their capacity to attract neutrophils and monocytes through CCL2 and CCL7 production, ultimately promoting tubular atrophy and fibrosis (35, 39). Future studies incorporating detailed phenotypic and functional analyses of these myeloid populations, alongside their interactions with B cells, will be essential to construct a complete mechanistic framework of post-splenectomy IRI progression. Second, the histological identification of TLTs is lacking. Organized lymphocyte aggregates, defined as TLTs (40, 41), were not specifically characterized in this study. The identification of KMNCs with periodic acid-Schiff (PAS) staining and assessment of the lymphocyte infiltrates using immunofluorescence markers, such as CD3ϵ, CD20, Ki67, and CD21, have been used in previous studies (23, 40). Third, plasma creatinine and cystatin C levels were used to estimate kidney function. However, plasma creatinine and cystatin C levels are not sensitive measures for assessing renal function in a unilateral IRI model because of compensation from the functional contralateral kidney (42). Further studies using more sensitive measures, such as transcutaneous GFR (43, 44), multispectral optoacoustic tomography (45), or biomarkers such as NGAL and KIM1, are required to assess renal function or injury more precisely. Additionally, since the assessment of fibrosis in our study was limited to histologic findings, future studies using gene expression analysis, including αSMA, collagen IV, and TGFβ1, are warranted to validate and strengthen these findings. Although we also analyzed the contralateral uninjured kidneys as controls, normal or sham control groups would have provided a clearer baseline for interpreting immunological changes following splenectomy and IRI. While our study demonstrates associations between splenectomy, B cell alterations, and worsened IRI outcomes, we acknowledge that causal mechanistic relationships remain to be established. Specifically, we cannot determine whether the observed cytokine alterations originate from expanded B cells or are induced by B cell-mediated activation of other cells. Our findings provide a foundation for future mechanistic studies employing B cell-specific interventions to definitively determine whether enhanced B cell responses directly drive the exacerbated injury observed in post-splenectomy IRI.

Despite these limitations, our study provides important insights into the impact of splenectomy on the repair phase of kidney IRI, highlighting the impact of splenectomy on B cell differentiation and its potential contribution to immune modulation in post-ischemic kidneys. Splenectomy is commonly used as a therapeutic strategy for benign hematological diseases, such as immune thrombocytopenia, hemolytic anemia, and Evans syndrome (46, 47). And the risk of kidney injury following splenectomy does exist (48). The findings from the present study suggest that close monitoring and nephroprotective care for patients who undergo splenectomy may be necessary to reduce the risk of AKI-to-CKD transition.

In conclusion, our study demonstrated that splenectomy facilitates the differentiation and activation of kidney B cells, ultimately impairing the healing process during the repair phase of kidney IRI. Contrary to the beneficial effects of splenectomy observed in the acute phase of AKI, splenectomy can impair the repair process in ischemic AKI, potentially leading to an incomplete recovery or progression to CKD. Exploring the reparative pathways of the spleen–kidney axis is a promising area for future research.

## Data Availability

The original contributions presented in the study are included in the article/**Supplementary Material**. Further inquiries can be directed to the corresponding author.
