# Supplementary material for: Splenectomy modulates intrarenal B cell differentiation and impairs repair of post-ischemic kidney
Source: Front Immunol. 2025 Nov 19;16:1684731. doi: 10.3389/fimmu.2025.1684731 (PMC12672880; doi:10.3389/fimmu.2025.1684731)
Supplement: Supplementary file 1 [file DataSheet1.docx]

## Supplementary Figures

**Supplementary Figure S1. Representative image of whole-slide CD45 immunohistochemistry analysis using QuPath.**

**
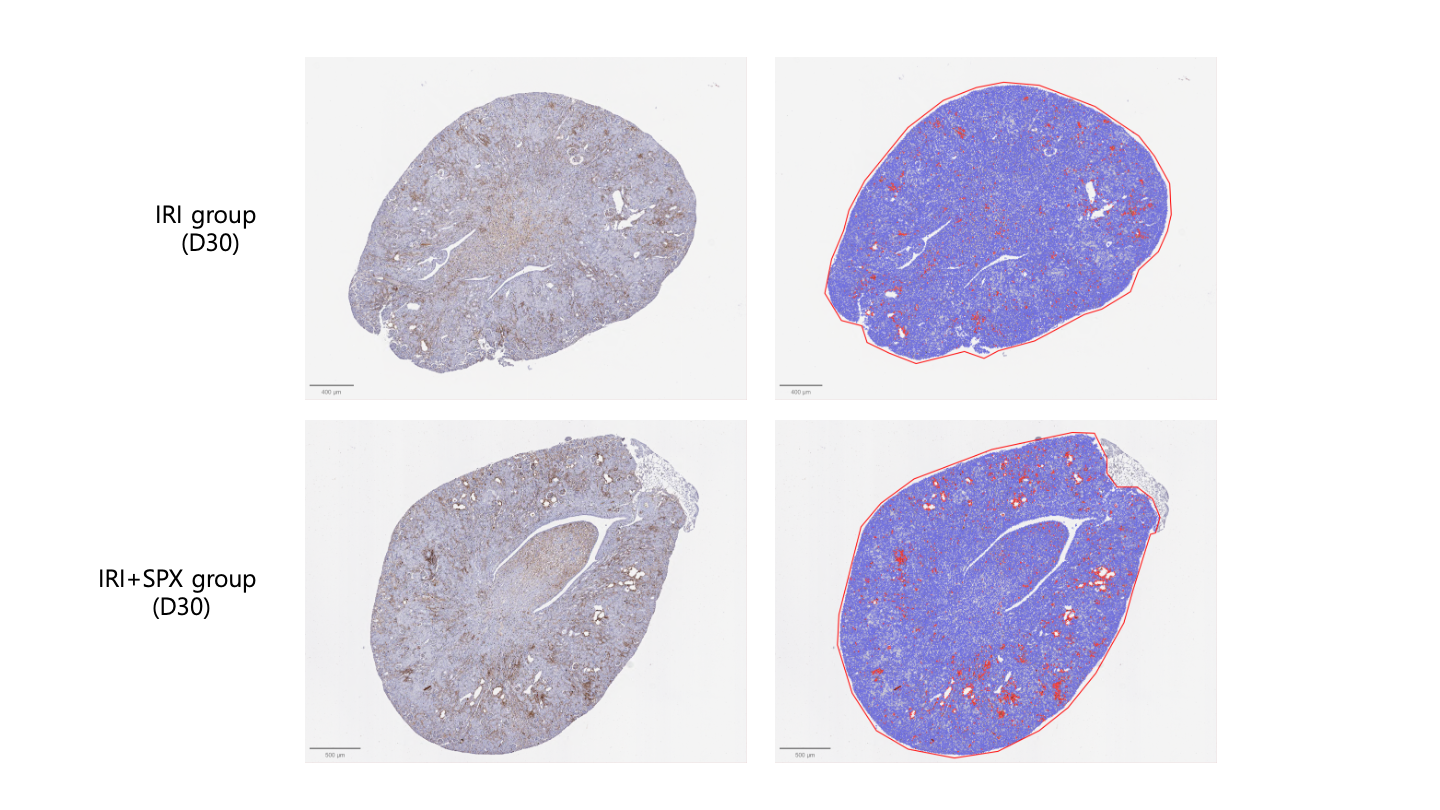
**

Post-ischemic kidney sections were stained for CD45 and scanned for digital analysis. Positive CD45+ cells (brown DAB staining) were automatically detected across the entire kidney section using standardized intensity thresholds in QuPath. The detection overlays indicate CD45+ cells (highlighted) relative to total nucleated cells identified by hematoxylin counterstaining. This method enabled objective and complete quantification of leukocyte infiltration throughout the cortex and outer medulla. All samples were analyzed under identical threshold settings to ensure consistency.

**Supplementary Figure S2. Flow cytometry gating strategy for the analysis of B cell populations.**

**
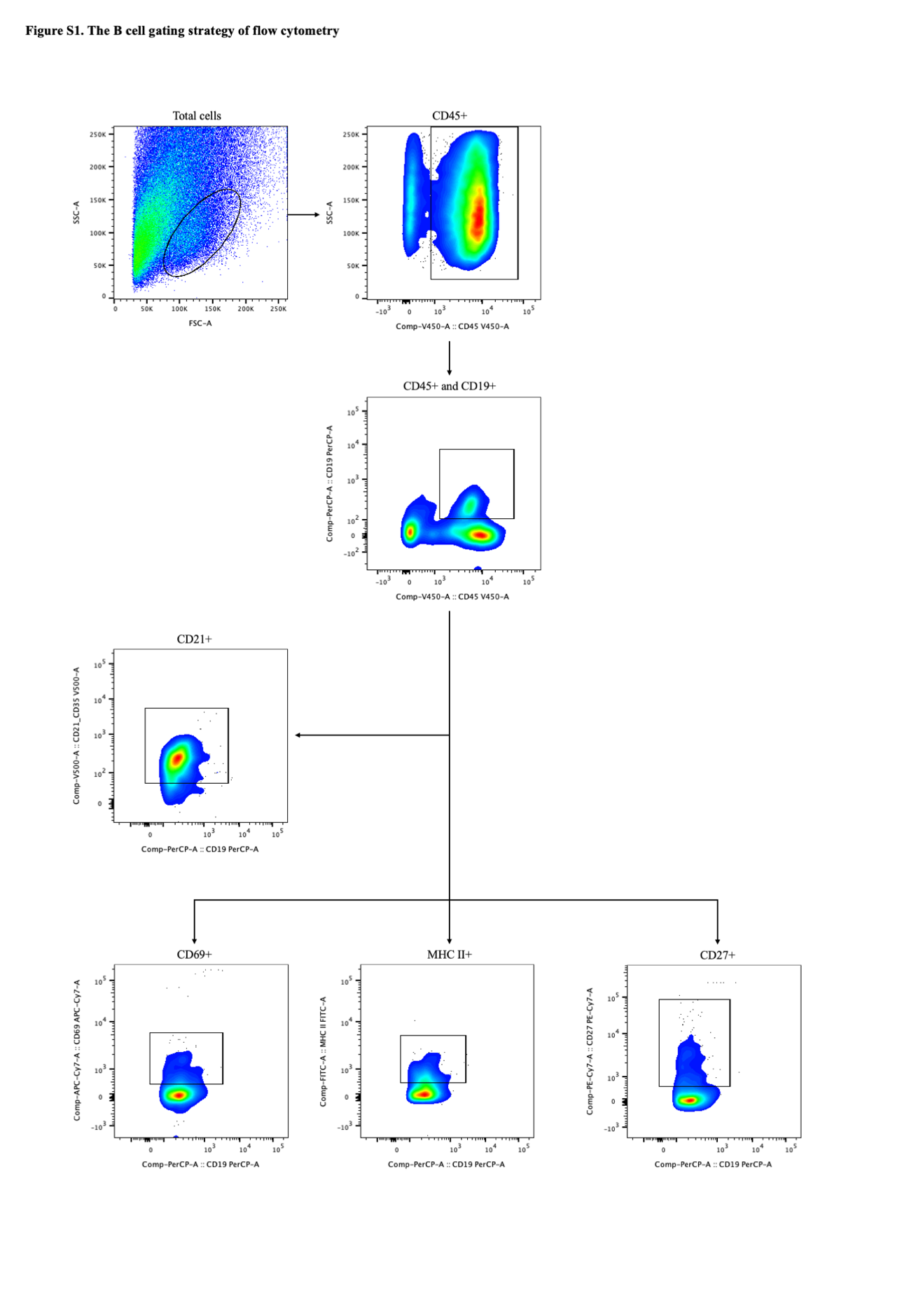
**

Single-cell suspensions were prepared from post-ischemic and contralateral kidneys. After initial gating on singlets and viable cells, CD45+ leukocytes were identified. B cells were defined as CD19+ cells, and further subcategorized into mature B cells (CD21+), activated B cells (CD69+), MHCII+ B cells, memory B cells (CD27+), and plasma cells (CD138+ and CD126+). Proper compensation controls and fluorescence minus one (FMO) controls were used to ensure accurate gating.

**Supplementary Figure S3. Flow cytometry gating strategy for the analysis of T cell populations.**


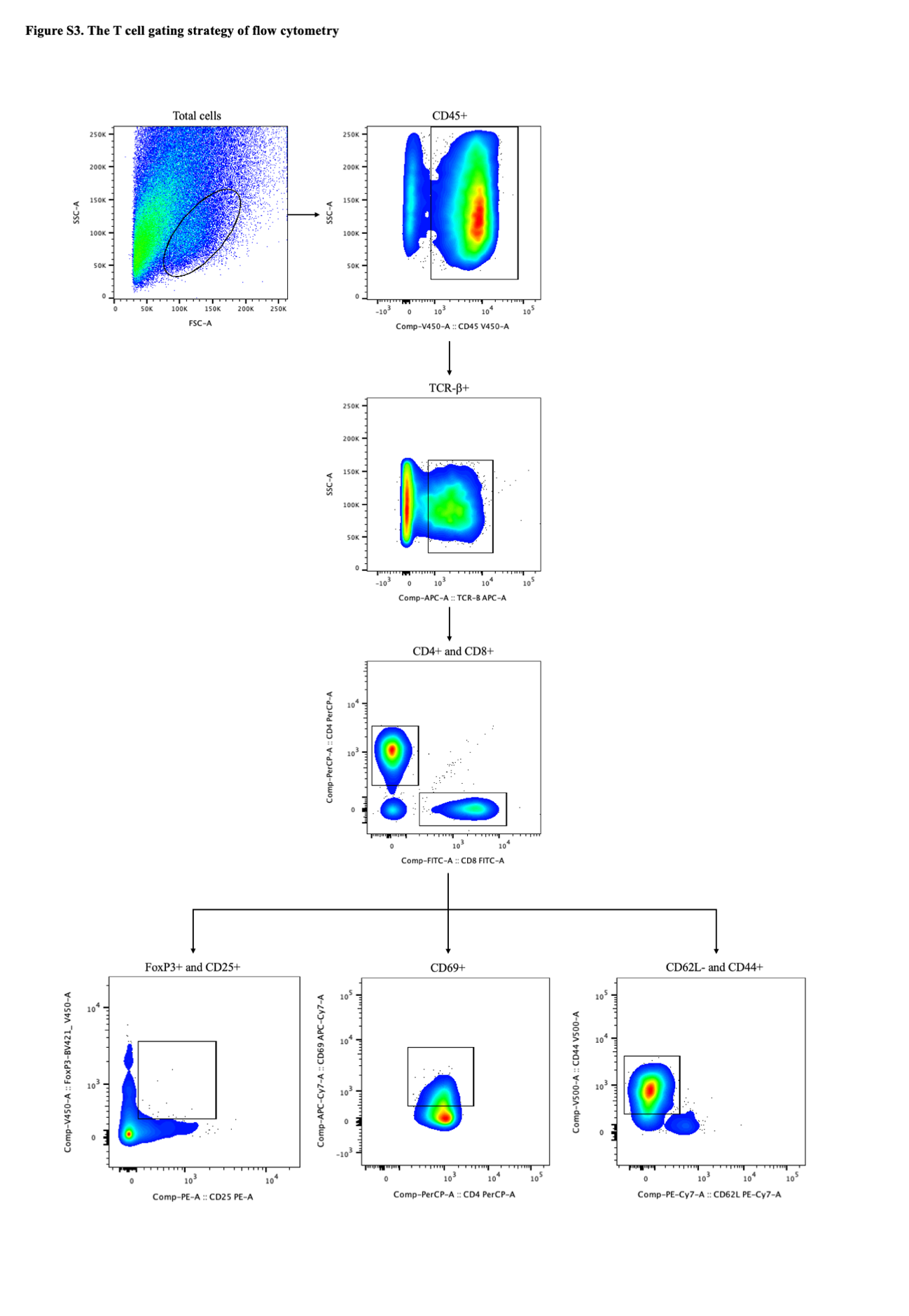


Single-cell suspensions were prepared from post-ischemic and contralateral kidneys. After initial gating on singlets and viable cells, T cells were identified as CD3+ cells, with subsequent gating for CD4+ and CD8+ T cell subsets. Regulatory T cells (Tregs) were defined as CD4+CD25+FoxP3+ cells. Within both CD4+ and CD8+ T cell compartments, activated T cells were defined as CD69+, and effector memory T cells were defined as CD62L-CD44+. Proper compensation controls and fluorescence minus one (FMO) controls were used to ensure accurate gating.

**Supplementary Figure S4. Analysis of T cell subtypes in post-ischemic and contralateral kidneys on days 10 and 30 post-IRI in the IRI and IRI+SPX groups.**


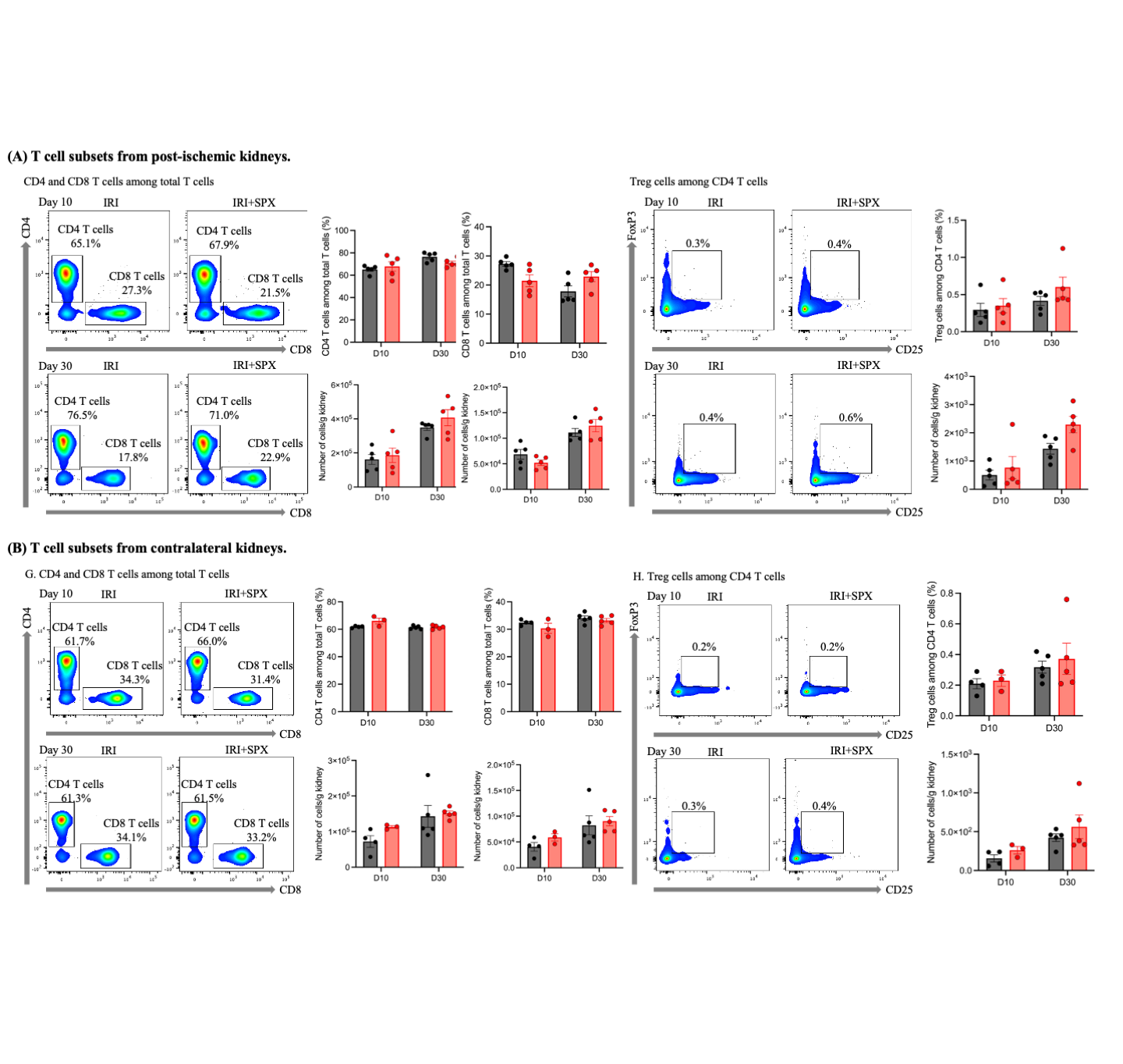


Flow cytometric analysis of T cell subsets in (A) post-ischemic and (B) contralateral kidneys. Single-cell suspensions were prepared from kidneys, and immune cell populations were analyzed by flow cytometry. T cell subsets included total T cells (CD3+), CD4+ T cells, CD8+ T cells, and regulatory T cells (Tregs; CD4+CD25+FoxP3+). Both percentages and absolute counts of T subsets were analyzed. While compositional differences were reflected in percentages, absolute cell counts provided complementary quantitative data for each subset. Measurements were taken on days 10 and 30 after IRI. Data are expressed as mean ± SD (n=5 per group). Statistical significance was assessed by Mann-Whitney U test. P < 0.05 was considered statistically significant.
