# Supplementary material for: Splenectomy modulates intrarenal B cell differentiation and impairs repair of post-ischemic kidney
Source: Front Immunol. 2025 Nov 19;16:1684731. doi: 10.3389/fimmu.2025.1684731 (PMC12672880; doi:10.3389/fimmu.2025.1684731)
Supplement: Supplementary file 2 [file Table1.docx]

## Supplementary Tables

**Supplementary Table S1. Absolute count of B cell populations in post-ischemic and contralateral kidneys**

| Number of cells/g kidney  in post-ischemic kidneys | Day 10 after IRI | | | Day 30 after IRI | | |
| --- | --- | --- | --- | --- | --- | --- |
|  | IRI | IRI+SPX | p-value | IRI | IRI+SPX | p-value |
| Total B cells | 271488±33436 | 289553±55327 | 0.690 | 78553±13321 | 49219±5898 | 0.032 |
| Activated B cells | 6012±1138 | 7647±1294 | 0.421 | 6648±503 | 10273±1504 | 0.016 |
| MHCII+ B cells | 9426±2582 | 8128±3466 | 0.999 | 3954±784 | 7939±2150 | 0.031 |
| Mature B cells | 144470±7066 | 99618±7476 | 0.547 | 72575±12839 | 38375±6520 | 0.032 |
| Memory B cells | 10163±1500 | 9663±1264 | 0.690 | 6196±453 | 9426±1581 | 0.031 |
| Plasma cells | 4732±1329 | 5547±1440 | 0.690 | 3331±697 | 3070±581 | 0.841 |
| Number of cells/g kidney  in contralateral kidneys | Day 10 after IRI | | | Day 30 after IRI | | |
|  | IRI | IRI+SPX | p-value | IRI | IRI+SPX | p-value |
| Total B cells | 278164±79438 | 205892±63104 | 0.008 | 1869610±30280 | 74175±3194 | 0.001 |
| Activated B cells | 5183±1300 | 5926±2208 | 0.999 | 2474±392 | 4556±167 | 0.008 |
| MHCII+ B cells | 19632±5586 | 20779±5788 | 0.690 | 14952±1991 | 23432±613 | 0.008 |
| Mature B cells | 257524±74421 | 179359±53864 | 0.547 | 178518±28647 | 65970±2872 | 0.001 |
| Memory B cells | 18565±12044 | 15356±10921 | 0.690 | 11778±3854 | 8834±1391 | 0.095 |
| Plasma cells | 1610±602 | 717±351 | 0.032 | 917±242 | 160±34.0 | 0.032 |

Values represent the mean ± SEM of percentages of gated cells; n = 5/group. KMNCs, kidney mononuclear cells (KMNCs) expressing CD45 of lymphocyte gating on the FSC vs. SSC plot; total B cells expressing CD19 among total KMNCs; activated B cells expressing both CD69 and CD21 among total B cells; MHC II+ B cells expressing MHC II among total B cells; mature B cells expressing CD21 among total B cells; memory B cells expressing CD27 among total B cells; and plasma cells expressing both CD138 and CD126 among total B cells.

IRI, ischemia-reperfusion injury; SPX, splenectomy.

**Supplementary Table S2. Absolute count of T cell populations in post-ischemic and contralateral kidneys**

| Number of cells/g kidney  in post-ischemic kidneys | Day 10 after IRI | | | Day 30 after IRI | | |
| --- | --- | --- | --- | --- | --- | --- |
|  | IRI | IRI+SPX | p-value | IRI | IRI+SPX | p-value |
| Total T cells | 110577±8249 | 110359±31525 | 0.222 | 692546±76405 | 710419±83398 | 0.999 |
| CD8 T cells | 68500±9620 | 51939±5374 | 0.309 | 111329±7935 | 124547±12013 | 0.309 |
| CD4 T cells | 161840±28950 | 186201±42697 | 0.690 | 349100±16750 | 407182±47190 | 0.548 |
| Treg cells | 505±170 | 769±393 | 0.690 | 1436±193 | 2292±289 | 0.056 |
| Number of cells/g kidney  in contralateral kidneys | Day 10 after IRI | | | Day 30 after IRI | | |
|  | IRI | IRI+SPX | p-value | IRI | IRI+SPX | p-value |
| Total T cells | 125797±27033 | 185980±3417 | 0.035 | 227084±72854 | 246200±10888 | 0.151 |
| CD8 T cells | 40451±8517 | 58679±6995 | 0.114 | 82257±18345 | 90355±8921 | 0.309 |
| CD4 T cells | 71946±16304 | 112822±3696 | 0.114 | 142586±30499 | 151039±6577 | 0.222 |
| Treg cells | 154±47.4 | 259±48.3 | 0.228 | 422±49.3 | 562±151 | 0.999 |

Values represent the mean ± SEM of percentages of gated cells; n = 5/group. KMNCs: kidney mononuclear cells (KMNCs) expressing CD45 of lymphocyte gating on the FSC vs. SSC plot; total T cells expressing CD3 among total KMNCs; CD8 T cells expressing CD8 among total T cells; CD4 T cells expressing CD4 among total T cells; regulatory T (Treg) cells expressing both FoxP3 and CD25 among total T cells.

IRI, ischemia-reperfusion injury; SPX, splenectomy.

**Supplementary Table S3. Subsets of T cell populations in post-ischemic and contralateral kidneys**

| % of cells  in post-ischemic kidneys | Day 10 after IRI | | | Day 30 after IRI | | |
| --- | --- | --- | --- | --- | --- | --- |
|  | IRI | IRI+SPX | p-value | IRI | IRI+SPX | p-value |
| Activated CD4 T cells | 14.04±1.13 | 15.68±1.91 | 0.690 | 31.04±4.74 | 31.70±1.84 | 0.547 |
| Activated CD8 T cells | 28.08±3.56 | 28.96±7.11 | 0.999 | 52.22±4.20 | 49.88±1.69 | 0.547 |
| Effector memory CD4 T cells | 81.58±2.73 | 80.48±3.39 | 0.794 | 89.24±0.46 | 88.48±1.48 | 0.309 |
| Effector memory CD8 T cells | 70.78±4.39 | 57.78±4.70 | 0.095 | 80.30±1.07 | 84.52±0.97 | 0.032 |
| % of cells  in contralateral kidneys | Day 10 after IRI | | | Day 30 after IRI | | |
|  | IRI | IRI+SPX | p-value | IRI | IRI+SPX | p-value |
| Activated CD4 T cells | 19.15±2.83 | 8.89±2.41 | 0.057 | 42.96±4.43 | 47.58±5.52 | 0.421 |
| Activated CD8 T cells | 12.30±3.22 | 6.88±1.93 | 0.229 | 26.60±3.88 | 37.32±5.96 | 0.310 |
| Effector memory CD4 T cells | 51.85±3.22 | 28.17±8.65 | 0.114 | 22.66±2.70 | 29.60±4.20 | 0.309 |
| Effector memory CD8 T cells | 28.33±4.56 | 13.58±2.97 | 0.057 | 14.57±2.50 | 19.89±3.47 | 0.198 |

Values represent the mean ± SEM of percentages of gated cells; n = 5/group. KMNCs: kidney mononuclear cells (KMNCs) expressing CD45 of lymphocyte gating on the FSC vs. SSC plot; activated T cells, identified as CD69⁺ cells within CD4+ or CD8+ subsets; effector memory T cells, defined as CD62L-CD44+ cells within CD4+ or CD8+ subsets.

IRI, ischemia-reperfusion injury; SPX, splenectomy.
